# Bidirectional Mendelian Randomization Study Identifies No Genetic Link Between Psoriasis and Diabetes

**DOI:** 10.1155/jdr/9917071

**Published:** 2025-03-10

**Authors:** Jing Li, Min Li, Shoufang Kong, Chunmei Zhong, Danting Sun, Lili Zhang

**Affiliations:** ^1^Department of Nutrition, The Affiliated Hospital of Qingdao University, Qingdao, China; ^2^Department of Dermatology, Qingdao Municipal Hospital, Qingdao, China; ^3^Department of Gynaecology, Qingdao Hiser Hospital Affiliated of Qingdao University (Qingdao Traditional Chinese Medicine Hospital), Qingdao, China; ^4^Department of Gynaecology, Affiliated Hospital of Qingdao University, Qingdao, China

**Keywords:** causality, diabetes, FinnGen, Mendelian randomization, psoriasis

## Abstract

Epidemiological studies proposed a bidirectional link between psoriasis (Ps) and diabetes mellitus (DM); their causal relationship remains inadequately explored. We obtained summary statistics of genome-wide association analyses for Type 1 diabetes mellitus (T1DM), Type 2 diabetes mellitus (T2DM), and Ps from individuals of European ancestry by accessing the UK Biobank and FinnGen datasets. Inverse-variance weighted (IVW) method was utilized as the primary method. Additional analyses included debiased IVW (dIVW), constrained maximum likelihood with model averaging, robust adjusted profile score, Mendelian randomization (MR)–Egger, weighted median, and weighted mode. Moreover, sensitivity tests were conducted, including Cochran's *Q*, MR pleiotropy residual sum, and outlier analyses. Eventually, bidirectional MR was conducted to examine the possibility of a causal link between Ps and DM. No significant causal associations were indicated between DM and Ps. Moreover, there was no causal link between Ps and T1DM. Although certain positive correlations were identified between Ps and T2DM, aggregate evidence remains insufficient to establish a causal relationship. The results demonstrated no evidence of horizontal pleiotropy between genetic variants. Furthermore, a leave-one-out test validated the stability and robustness of this correlation. Our study identifies no genetic causal effect of Ps on DM and of DM on Ps in European ancestry. Additional research is warranted to verify the presence of an association between Ps and DM in diverse populations.

## 1. Introduction

Current research has established that psoriasis (Ps) is a systemic inflammatory disease related to several comorbidities, including metabolic disorders, gastrointestinal conditions, renal impairments, malignancies, and infections [1]. Diabetes mellitus (DM) represents a metabolic disease defined by persistent hyperglycemia attributed to insulin resistance (IR) or deficiency, which contributes to microvascular and macrovascular complications [2, 3]. The pathogenesis of Ps and DM involves complex dysregulation of inflammatory response and autoimmune processes, and their association has increasingly attracted widespread attention. Inflammatory cytokines, including tumor necrosis factor, present in individuals with DM may cause the onset of Ps [4]. Studies have shown that Ps occurs more frequently among individuals diagnosed with DM than among the general population [5]. At the same time, Ps-related chronic inflammation can promote IR and is related to the pathogenesis of Type 2 diabetes mellitus (T2DM) [6–8]. Some studies have found that Ps is related to the increased risk of diabetes. With the increase in the duration and severity of Ps, the risk of diabetes and metabolic syndrome will increase [4, 9, 10]. However, the causal relationship between DM and Ps remains controversial. A prospective cohort study found that diabetes was not associated with Ps risk [11]. At the same time, cross-sectional studies have also found that Ps is unrelated to the risk of diabetes [12]. At present, the causal relationship between DM and Ps is not fully understood. The above observational studies have limitations, such as a lack of randomness and large sample data support, and are susceptible to confounding factors, rendering them insufficient for establishing causal relationships. Further evidence independent of potential confounding variables is needed to validate the causal relationship between DM and Ps. Mendelian randomization (MR) studies utilize genetic variants (GVs), including single nucleotide polymorphisms (SNPs), as instrumental variables (IVs) to divide participants into distinct groups unaffected by confounding factors [13]. Genetic variations remain unaffected by environmental influences and other genetic factors, thereby minimizing the impact of confounding variables. These variations are established prior to birth and remain unaltered by disease processes, which reduces the potential for reverse causality [14]. Two-way MR is a methodological approach employed to investigate the bidirectional relationship between exposures and outcomes through two independent MR analyses, facilitating the determination of causal directionality. This approach is particularly advantageous for examining scenarios where bidirectional causal relationships may exist.

## 2. Materials and Methods

### 2.1. GVs Associated With DM and Ps

**Figure 1:**
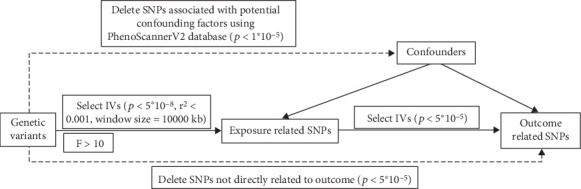
Flowchart of the study.

SNP-based genome-wide association analyses (GWAS) for Type 1 diabetes mellitus (T1DM) and T2DM were acquired by accessing the IEU OpenGWAS project (https://gwas.mrcieu.ac.uk/), which performed a meta-analysis incorporating data from the UK Biobank and FinnGen studies [9–11]. Moreover, we obtained the most recent data for T1DM and T2DM from the FinnGen study (https://r10.finngen.fi/) [15–17]. Meanwhile, we acquired GWAS data for Ps by accessing the FinnGen study and the UK Biobank dataset using SAIGE analysis (https://pheweb.org/UKB-SAIGE/) [18]. Details are presented in Table 1. These datasets underwent MR analysis, satisfying three fundamental assumptions (Figure 1): relevance, independence, and exclusion restriction [19]. To fulfill these assumptions, exposure-related IVs were required to be correlated and highly correlated with a *p* value < 5 × 10^−8^, within a 1 Mb distance, and an *r*-squared value of < 0.001. The *F*-statistics of SNPs were calculated as follows: *F*‐statistic = (beta/Se)2 [20, 21], with a threshold of *F* > 10 employed to address potential bias arising from weak IVs [22]. The conditions for outcome-related IVs included a *p* value threshold of <5 × 10^–5^. More detailed information on participant selection and data processing can be found in original articles and websites. The ethical approval and consent information for these data can be found in the original publication.

### 2.2. Univariable MR

We performed a Wald ratio to assess the effect of exposure on an outcome for each genetic instrument. Following harmonization and IV selection, we conducted MR analyses employing the MR R package (Version 0.5.6) within the R software environment (Version 4.3.0). Inverse-variance weighted (IVW) method was implemented to provide a weighted average of the causal effect estimates [23]. The primary MR approach utilized in our study was the two-sample IVW method, chosen because of the fulfillment of MR assumptions by all IVs [23]. In cases of significant heterogeneity, we utilized a random-effects IVW model as the primary analysis method; otherwise, we deployed a fixed-effects IVW model [24]. The debiased IVW (dIVW) method eliminates the weak instrument bias of the IVW method and is more robust in the presence of weak instruments [25, 26]. MR-Egger (MRE) regression can detect horizontal pleiotropy (HP) through its intercept and generate an estimate adjusted for pleiotropic effects; however, it has less statistical power [27]. Constrained maximum likelihood with model averaging (cML-MA) is used to exclude biases caused by related and unrelated pleiotropy. This method is considered much more powerful than MRE [28]. The MR-RAPS method allows for the inclusion of some weak IVs, enabling robust statistical estimation of MR using these weak IVs. When considering special pleiotropy, it can provide robust inference for MR analyses involving many weak IVs [29]. The weighted median (WME) can provide consistent estimates even when some of the GVs in the analysis are not valid IVs [30]. Weighted mode (WMO) has the power to detect a causal effect that is smaller than that of the IVW and WME methods but larger than that of the MRE regression [31]. We performed bidirectional MR analysis: the forward MR was to explore the effect of DM on Ps, and the reverse MR was to explore the effect of Ps on DM. Cochran's *Q* test was utilized to detect heterogeneity [32]. Furthermore, the MRE regression method was utilized to assess the potential presence of HP [33]. Moreover, we utilized a funnel plot to evaluate the likely directionality of pleiotropy [34]. Our study performed a leave-one-out analysis to determine the effect of individual SNPs on the results (Figure S1) [27]. Our study employed a significance level of 0.05 for hypothesis testing. The results of sensitivity analyses are summarized in Table S13.

### 2.3. GV Selections and Sensitivity Analysis

Palindromic SNPs were excluded from the analysis. The sensitivity analysis included heterogeneity, pleiotropy, and leave-one-out sensitivity tests. Cochran's *Q* test was utilized to detect heterogeneity [32]. HP is commonly present in causal relationships between complex traits and diseases. When employing genetic instruments, there is a potential for HP, where a GV influences an outcome through a biological pathway that is independent of the exposure, potentially introducing bias [33]. MR pleiotropy residual sums and outlier (MR-PRESSO) analyses identified pleiotropy outliers across multiple IV summary levels and were employed to detect and exclude outlier instruments, thereby mitigating the impact of HP [33]. The MRE method accounts for the influence of pleiotropy in various settings, providing estimates that are corrected for pleiotropy and thereby enhancing the accuracy of causal estimation [33]. To mitigate HP, candidate IVs were identified in the PhenoScannerV2 database (http://www.phenoscanner.medschl.cam.ac.uk/) based on their association with potential confounding factors (*p* < 5 × 10^−8^) [34]. To investigate the relationship between DM and Ps, confounding factors that included alcohol intake, body mass index (BMI), and past tobacco smoking were considered. Although DM was set as the outcome, BMI was a confounding factor. Moreover, our study performed a leave-one-out analysis to determine the effect of individual SNPs on the results (Figure S1) [28]. Our study employed a significance level of 0.05 for hypothesis testing. Supplementary Tables S1–12 list the details of the SNPs. The results of sensitivity analyses are summarized in Table S13. The research process is shown in Figure 1.

## 3. Results

### 3.1. The Causal Impact of T1DM on Ps

**Figure 2:**
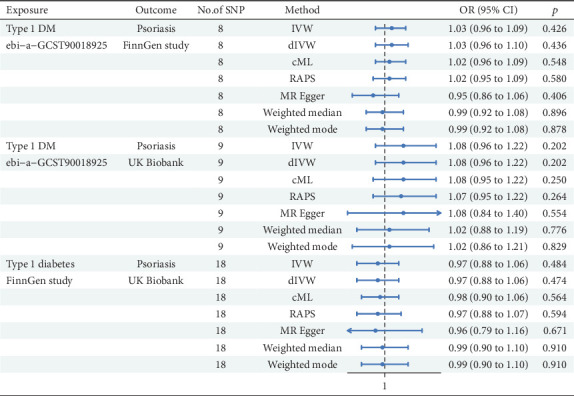
MR analysis from Type 1 diabetes to psoriasis.

An MR analysis was deployed to investigate the probable causal correlation between T1DM and Ps (Figure 2). For the T1DM as exposure from OpenGWAS and Ps as outcome from the FinnGen study (nSNP = 8), the IVW model indicated no statistically significant link between T1DM and Ps (*β* = 0.026, 95%CI = 1.03 (0.96, 1.09); *p* = 0.426). Comparable nonsignificant results were observed for the dIVW, cML, robust adjusted profile score (RAPS), MRE, WME, and WMO methods. When analyzing T1DM data from OpenGWAS and Ps data from the UK Biobank (nSNP = 9), the IVW model again demonstrated no significant link (*β* = 0.079, 95%CI = 1.08 (0.96, 1.22); *p* = 0.202). The results of the dIVW, cML, RAPS, MRE, WME, and WMO methods were consistent with IVW findings. The analysis of T1DM from FinnGen and Ps from UK Biobank data (nSNP = 18) revealed that the IVW model manifested a nonsignificant association (*β* = –0.034, 95%CI = 0.967 (0.879, 1.063); *p* = 0.484). The dIVW, cML, and RAPS methods were consistent with IVW results. The MRE, WME, and WMO methods all indicated no significant causal relationship. In conclusion, MR analysis of T1DM to Ps did not offer compelling evidence for a statistically significant causal link.

### 3.2. The Causal Impact of T2DM on Ps

**Figure 3:**
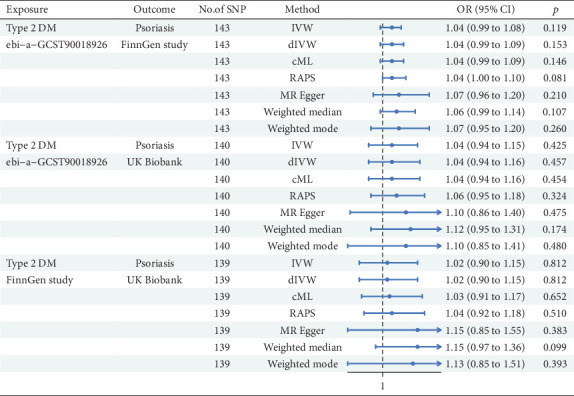
MR analysis from Type 2 diabetes to psoriasis.

As shown in Figure 3, the FinnGen analysis of Ps examined 143 SNPs, and the IVW method demonstrated that T2DM had a positive but not statistically significant association with Ps (*β* = 0.035, 95%CI = 1.04 (0.99, 1.08); *p* = 0.119). The dIVW method yielded similar results as well as did the cML method. The RAPS represented a trend toward significance. The MRE test did not reach statistical significance. The WME and WMO methods also implied a positive relationship; however, the relationship exhibited no statistical significance. The FinnGen analysis of Ps that evaluated 140 SNPs implied that the IVW model elucidated a nonsignificant association (*β* = 0.040, 95%CI = 1.04 (0.94, 1.15); *p* = 0.425). The dIVW model mirrored these findings. The cML method indicated a slightly stronger association, but it showed no statistical significance. The RAPS approached significance. The MRE method failed to support a significant causal effect. Both WME and WMO methods exhibited similar trends. In the analysis of T2DM from FinnGen and Ps from the UK Biobank data (nSNP = 139), the IVW method revealed no significant link (*β* = 0.015, 95%CI = 1.02 (0.90, 1.15); *p* = 0.812). The dIVW method was consistent with IVW findings. The cML method indicated a positive association but with no statistical significance. The RAPS manifested a similar trend. The MRE method failed to reach statistical significance. Both WME and WMO methods suggested a positive relationship, although statistical significance was not achieved. Altogether, our MR study revealed no statistically significant causal link between T2DM and Ps across the evaluated methods and data sources.

### 3.3. The Causal Impact of Ps on T1DM

**Figure 4:**
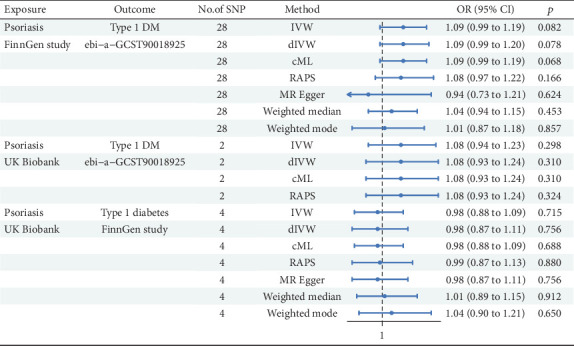
MR analysis from psoriasis to Type 1 diabetes.

Figure 4 presents the results of an MR study designed to explore the potential causal correlation between Ps and T1DM.

In Ps from FinnGen analysis (nSNP = 28), the IVW method proposed that Ps had a positive association with T1DM (*β* = 0.083, 95%CI = 1.09 (0.99, 1.19); *p* = 0.082). Both dIVW and cML methods aligned with the IVW results. Nonetheless, the RAPS exhibited no statistical significance. The MRE method showed a negative but nonsignificant association. The WME and WMO methods did not indicate significant associations. In Ps from the UK Biobank analysis (nSNP = 2), the IVW method displayed no significant link (*β* = 0.073, 95%CI = 1.08 (0.94, 1.23); *p* = 0.298). The dIVW, cML, and RAPS methods mirrored these findings. In the analysis of T1DM from FinnGen and Ps from UK Biobank data (nSNP = 4), the IVW method showcased a nonsignificant association (*β* = −0.020, 95%CI = 0.98 (0.88, 1.09); *p* = 0.715). The dIVW and cML methods were consistent with IVW results. The RAPS and MRE method results indicated no significant association. Both WME and WMO methods failed to support the existence of a significant causal relationship.

The MR analysis from Ps to T1DM across the FinnGen study and the UK Biobank did not yield statistically significant evidence for a causal relationship. This indicates that Ps may not be causally linked to the incidence of T1DM in the study population.

### 3.4. The Causal Impact of Ps on T2DM

**Figure 5:**
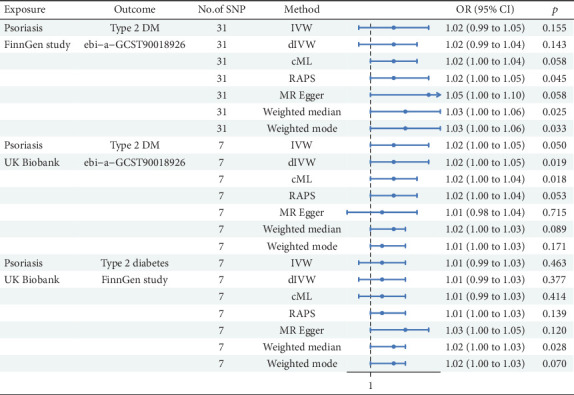
MR analysis from psoriasis to Type 2 diabetes.

Our study utilized MR analysis to investigate the possible causal effect of T2DM on Ps utilizing data from the FinnGen study and the UK Biobank, as shown in Figure 5. With 31 SNPs assessed for Ps in the FinnGen study as exposure, the IVW method elucidated a small but nonsignificant positive link between T2DM and Ps (*β* = 0.018, 95%CI = 1.02 (0.99, 1.05); *p* = 0.155). The dIVW method also manifested a nonsignificant positive relation. The cML method suggested a marginally significant positive association. The RAPS indicated a stronger positive association, approaching significance. The MRE analysis also indicated a positive association, which was of borderline significance. Both WME and WMO methods indicated positive associations, with the WME being significant. In the Ps of UK Biobank as exposure (nSNP = 7), the IVW method manifested a borderline significant positive link (*β* = 0.024, 95%CI = 1.02 (1.00, 1.05); *p* = 0.050). The dIVW method mirrored these findings with a nonsignificant result. The cML method demonstrated a significantly positive correlation. The RAPS showcased a positive association, albeit not significant. The MRE test indicated no significant association. Both WME and WMO methods showed nonsignificant positive associations. In the analysis of T2DM from FinnGen as outcome and Ps as exposure from UK Biobank data, the IVW method did not indicate a significant association (*β* = 0.008, 95%CI = 1.01 (0.99, 1.03); *p* = 0.463). The dIVW and cML methods were consistent with IVW results. The RAPS elucidated a borderline significant positive correlation. The MRE test failed to provide evidence of a significant link. Both WME and WMO methods indicated positive associations, with the WME being significant.

The MR analysis from T2DM to Ps across the FinnGen study and the UK Biobank yielded mixed evidence regarding a causal relationship. Although some methods and data sources suggested a positive association, the majority did not reach statistical significance. However, the overall evidence is not insufficiently robust to establish a definitive causal link, and further research with larger sample sizes and additional data sources is warranted.

The FinnGen study and the UK Biobank dataset consistently yielded results that demonstrate no statistically significant association between T1DM and Ps, T2DM and Ps, or Ps and T1DM. None of the methods employed (IVW, dIVW, cML, RAPS, MRE, WME, or WMO) established a significant causal relationship. Although there are some positive associations between Ps and T2DM, overall evidence is inadequate to definitively demonstrate a clear causal relationship.

### 3.5. Sensitivity Analysis

Our sensitivity analyses did not identify significant evidence of pleiotropy. The MRE intercept was not significantly different from zero, suggesting no substantial HP. Additionally, the leave-one-out analysis did not reveal any single SNP that significantly influenced the overall estimate, indicating that our results were robust and not driven by pleiotropic SNPs. The PRESSO analysis also supported the absence of significant outliers or pleiotropic SNPs.

## 4. Discussion

Currently, the association between Ps and DM has aroused widespread interest. According to a meta-analysis, patients with T2DM had a 27% heightened risk of Ps, whereas those with T1DM had a 43% increased risk [4]. Similarly, a systematic review revealed that patients having severe Ps had a 1.5-fold increased risk of developing T2DM, and even higher risks were observed among younger patients and those with severe Ps [35, 36]. This relationship may be a commonality between the two regarding pathogenesis, including being driven by chronic inflammation. Patients with Ps have been shown to have endogenous IR, which belongs to the most important causes of DM [6–8]. The contradictory evidence between our results and those of earlier observational studies on the relationship between Ps and DM may be owing to the following factors. First, the potential limitations of observational research, including bias related to selection, information, and misclassification, as well as confounding and statistical errors, were difficult to remove completely. The common risk factors for Ps and DM may have led to these observational studies being affected by these confounding factors, and the causal relationship between them cannot be confirmed. Obesity, inflammation, and genetic factors are associated with the pathogenesis of DM and Ps [37, 38]. These common risk factors may drive the observed association between the two diseases rather than one disease directly leading to the other. The diagnostic criteria and severity of diseases included in different studies may vary, which to some extent affects the credibility of the research results. Our study decreased the likelihood of unmeasured confounding factors that could reverse causal relationships in observational analyses and offered innovative insights into the causality between Ps and DM. For the first time, we thoroughly examined the causal relation between DM and Ps utilizing an MR analysis. In comparison to earlier observational studies, MR analysis using a GWAS dataset ensured the persistence of research results within the same ancestral population. This enhanced the credibility of the research results and the validity of the conclusions. Simultaneously, a sensitivity analysis was performed to improve the reliability of our findings. Another important consideration is the probable effect of DM or Ps therapy on the occurrence of another disease. Studies have disclosed that using corticosteroids and other drugs to manage Ps may increase the risk of DM [39]. Our analysis was unaffected by these therapeutic factors and can better evaluate the true causal relationship between DM and Ps. Our research failed to provide evidence supporting causal effects, suggesting that other factors, confounding variables, or biases may have contributed to the observed associations.

This study has several advantages. First, as a two-way MR study, it explored the potential causal relation between Ps and DM in both directions, offering deeper insights into their interplay. Second, it employed multiple MR analysis methods, including IVW, dIVW, cML-MA, RAPS, MRE, WME, and WMO, to ensure the robustness and reliability of the findings.

Nevertheless, our study has certain constraints. First, we used data derived exclusively from the European population, raising concerns about generalizing the results to other populations. Second, while the sensitivity analysis failed to elucidate any pleiotropy, the possibility of HP cannot be entirely excluded. Last, owing to the utilization of summary statistics from GWAS, the study lacks individual-level data, which may limit the depth of the analysis. Additional research is warranted to verify the presence of an association between Ps and DM in diverse populations and explore the possible mechanisms behind this relationship.

Our study possesses several methodological strengths that contribute to the field of research on Ps and DM. Notably, it is a bidirectional MR investigation, which allows for a comprehensive exploration of the potential causal relationship between Ps and DM in both directions, thereby providing a more nuanced understanding of their interplay. We employed a variety of MR methods, including IVW, dIVW, cML-MA, RAPS, MRE, WME, and WMO. Each of these methods serves a distinct purpose: IVW provides a straightforward weighted average of causal effect estimates under the assumption of no pleiotropy; dIVW corrects for the potential bias from weak instruments; cML-MA addresses both vertical and HP; RAPS offers robust inference in the presence of HP; MRE is sensitive to HP; WME is resistant to outliers and pleiotropic effects; and WMO is another approach that minimizes the impact of pleiotropic variants. The selection of these diverse methods was strategic to ensure the robustness and reliability of our findings, as each offers a different perspective on the potential causality, and their convergence or divergence can provide valuable insights into the validity of the observed associations.

However, our study is not without limitations. The data used were exclusively derived from individuals of European ancestry, which limits the generalizability of our results to other populations. This underscores the need for future research to include diverse populations to verify the association between Ps and DM and to explore potential population-specific effects. Similar studies in populations from different geographic regions and ethnic backgrounds were needed to assess the generalizability of our results. Additionally, while our sensitivity analyses did not identify significant pleiotropy, we acknowledge that the possibility of HP cannot be entirely dismissed, given the inherent limitations of MR studies, such as population specificity and weak instrument bias. Lastly, the use of summary statistics from GWAS precluded access to individual-level data, which may have constrained our analysis. Future research with individual-level data could provide a more in-depth understanding of the genetic pathways involved in the Ps-DM relationship. In light of these considerations, we suggest that cross-population studies and investigations into specific genetic mechanisms are warranted to elucidate the nature of the Ps-DM association further.

## 5. Conclusions

The MR studies failed to support the presence of a causal link between Ps and DM or between DM and Ps. Additional research is warranted to verify the presence of genetic association between Ps and DM in diverse populations.

## Figures and Tables

**Table 1:** The basic characters of selected traits.

**Trait**	**IEU OpenGWAS ID/source**	**Years**	**Population**	**Sample size**	**Case**	**Control**
Psoriasis	FinnGen study	2023	European	407,876	10,312	397,564
Psoriasis	UK Biobank	2021	European	400,436	2237	398,199
Type 1 diabetes	ebi-a-GCST90018925	2021	European	457,695	6447	451,248
Type1 diabetes	FinnGen study	2023	European	339,432	4320	335,112
Type 2 diabetes	ebi-a-GCST90018926	2021	European	490,089	38,841	451,248
Type 2 diabetes	FinnGen study	2023	European	400,197	65,085	335,112

## Data Availability

The original contributions presented in the study are included in the article or Supporting Information; further inquiries can be directed to the corresponding author.

## Supporting Information

Supporting Information 1 Table S1. Comprehensive data on the instrumental variables employed in the Mendelian randomization of Type 1 diabetes on psoriasis (ebi-a-GCST90018925 to finngen_R10_L12_PSORIASIS). Table S2. Comprehensive data on the instrumental variables employed in the Mendelian randomization of Type 1 diabetes on psoriasis (ebi-a-GCST90018925 to ukb-saige-696.4). Table S3. Comprehensive data on the instrumental variables employed in the Mendelian randomization of Type 1 diabetes on psoriasis (finngen_R10_T1D to ukb-saige-696.4). Table S4. Comprehensive data on the instrumental variables employed in the Mendelian randomization of Type 2 diabetes on psoriasis (ebi-a-GCST90018926 to finngen_R10_L12_PSORIASIS). Table S5. Comprehensive data on the instrumental variables employed in the Mendelian randomization of Type 2 diabetes on psoriasis (ebi-a-GCST90018926 to ukb-saige-696.4). Table S6. Comprehensive data on the instrumental variables employed in the Mendelian randomization of Type 2 diabetes on psoriasis (finngen_R10_T2D to ukb-saige-696.4). Table S7. Comprehensive data on the instrumental variables employed in the Mendelian randomization of psoriasis on Type 1 diabetes (finngen_R10_L12_PSORIASIS to ebi-a-GCST90018925). Table S8. Comprehensive data on the instrumental variables employed in the Mendelian randomization of psoriasis on Type 1 diabetes (ukb-saige-696.4 to ebi-a-GCST90018925). Table S9. Comprehensive data on the instrumental variables employed in the Mendelian randomization of psoriasis on Type 1 diabetes (ukb-saige-696.4 to finngen_R10_T1D). Table S10. Comprehensive data on the instrumental variables employed in the Mendelian randomization of psoriasis on Type 2 diabetes (finngen_R10_L12_PSORIASIS to ebi-a-GCST90018926). Table S11. Comprehensive data on the instrumental variables employed in the Mendelian randomization of psoriasis on Type 2 diabetes (ukb-saige-696.4 to ebi-a-GCST90018926). Table S12. Comprehensive data on the instrumental variables employed in the Mendelian randomization of psoriasis on Type 2 diabetes (ukb-saige-696.4 to finngen_R10_T2D). Table S13. Assessing pleiotropy and heterogeneity through MR-Egger intercept and Cochran's *Q* test.

Supporting Information 2 Figure S1. Leave-one-out plot of the causal relationships between diabetes and psoriasis. (A) Type 1 diabetes (ebi-a-GCST90018925) to psoriasis (FinnGen study); (B) Type 1 diabetes (ebi-a-GCST90018925) to psoriasis (UK Biobank); (C) Type 1 diabetes (FinnGen study) to psoriasis (UK Biobank); (D) Type 2 diabetes (ebi-a-GCST90018926) to psoriasis (FinnGen study); (E) Type 2 diabetes (ebi-a-GCST90018926) to psoriasis (UK Biobank); (F) Type 2 diabetes (FinnGen study) to psoriasis (UK Biobank); (G) psoriasis (FinnGen study) to Type 1 diabetes (ebi-a-GCST90018925); (H) psoriasis (UK Biobank) to Type 1 diabetes (ebi-a-GCST90018925); (I) psoriasis (FinnGen study) to Type 2 diabetes (ebi-a-GCST90018926); (J) psoriasis (UK Biobank) to Type 2 diabetes (ebi-a-GCST90018926); (L) psoriasis (UK Biobank) to Type 2 diabetes (FinnGen study).
