# Supplementary figures and images for: Bidirectional Mendelian Randomization Study Identifies No Genetic Link Between Psoriasis and Diabetes
Source: J Diabetes Res. 2025 Mar 10;2025:9917071. doi: 10.1155/jdr/9917071 (PMC11986917; doi:10.1155/jdr/9917071)

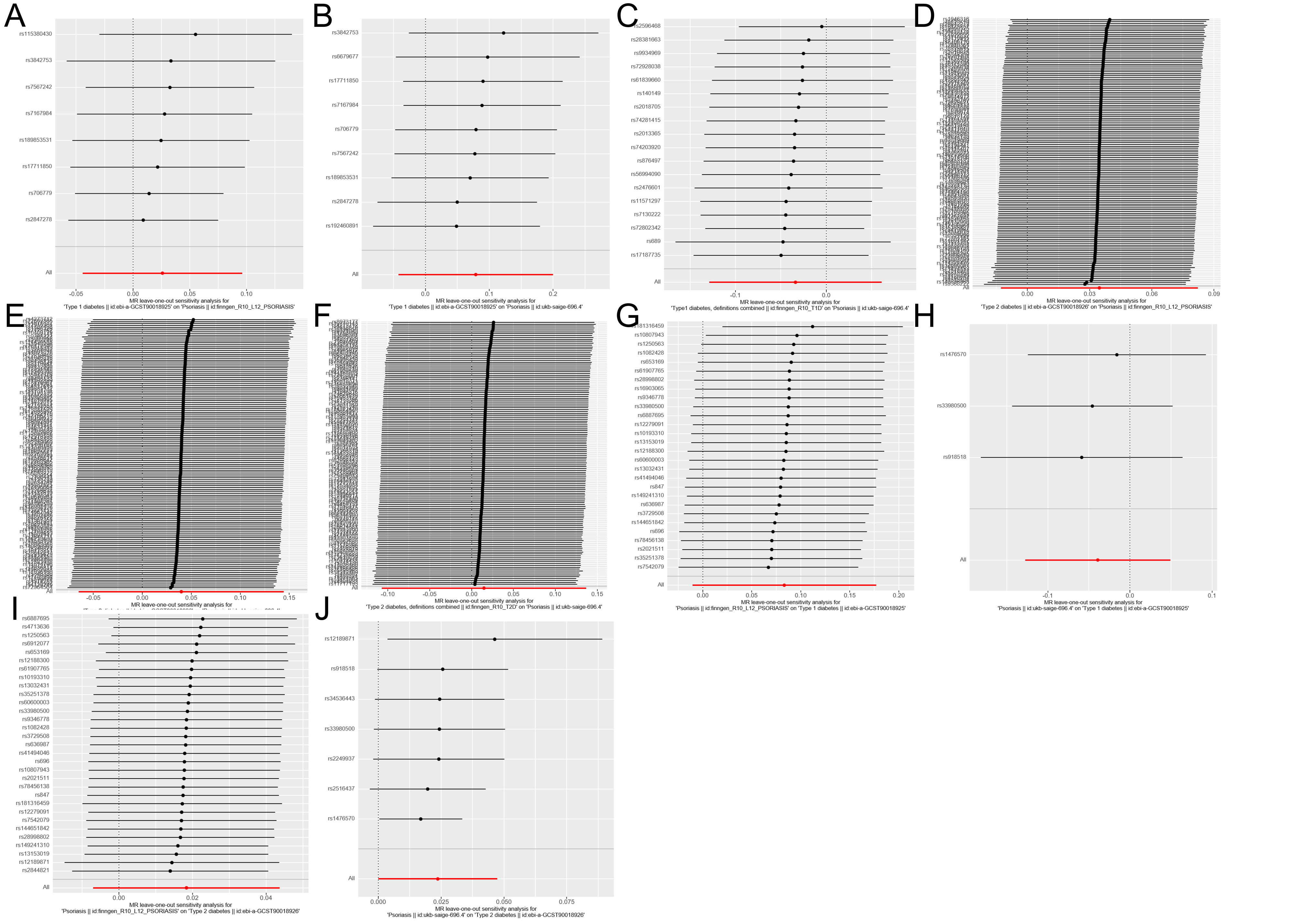

Supplement: Supporting Information 2 — Figure S1. Leave-one-out plot of the causal relationships between diabetes and psoriasis. (A) Type 1 diabetes (ebi-a-GCST90018925) to psoriasis (FinnGen study); (B) Type 1 diabetes (ebi-a-GCST90018925) to psoriasis (UK Biobank); (C) Type 1 diabetes (FinnGen study) to psoriasis (UK Biobank); (D) Type 2 diabetes (ebi-a-GCST90018926) to psoriasis (FinnGen study); (E) Type 2 diabetes (ebi-a-GCST90018926) to psoriasis (UK Biobank); (F) Type 2 diabetes (FinnGen study) to psoriasis (UK Biobank); (G) psoriasis (FinnGen study) to Type 1 diabetes (ebi-a-GCST90018925); (H) psoriasis (UK Biobank) to Type 1 diabetes (ebi-a-GCST90018925); (I) psoriasis (FinnGen study) to Type 2 diabetes (ebi-a-GCST90018926); (J) psoriasis (UK Biobank) to Type 2 diabetes (ebi-a-GCST90018926); (L) psoriasis (UK Biobank) to Type 2 diabetes (FinnGen study). [file 9917071.f2.tiff]
